# Modulating the Human Gut Microbiota through Hypocaloric Balanced Diets: An Effective Approach for Managing Obesity

**DOI:** 10.3390/nu15143101

**Published:** 2023-07-11

**Authors:** Hongchao Wang, Wenyan Song, Weiwei Yuan, Qunyan Zhou, Faizan Ahmed Sadiq, Jianxin Zhao, Wenjun Wu, Wenwei Lu

**Affiliations:** 1 State Key Laboratory of Food Science and Resources, Jiangnan University, Wuxi 214122, China; hcwang@jiangnan.edu.cn (H.W.); 6210113076@stu.jiangnan.edu.cn (W.S.); wer_yuan@163.com (W.Y.); zhaojianxin@jiangnan.edu.cn (J.Z.); 2 School of Food Science and Technology, Jiangnan University, Wuxi 214122, China; 3 Department of Nutriology, The Affiliated Wuxi People’s Hospital of Nanjing Medical University, Wuxi 214023, China; qunyan224@163.com; 4 Technology and Food Science Unit, Flanders Research Institute for Agriculture, Fisheries and Food (ILVO), 9090 Melle, Belgium; faizanahmed.sadiq@ilvo.vlaanderen.be; 5 Department of Endocrinology, The Affiliated Wuxi People’s Hospital of Nanjing Medical University, Wuxi 214023, China

**Keywords:** gut microbiota, dietary pattern, hypocaloric balanced diet, obesity, machine learning

## Abstract

This study aimed to investigate the effects of a hypocaloric balanced diet (HBD) on anthropometric measures and gut microbiota of 43 people with obesity. Fecal samples were collected from the study subjects at weeks 0 and 12, and a detailed analysis of gut microbiota was performed using 16S rRNA gene sequencing. By comparing anthropometric measures and microbiota changes in subjects before and after the HBD intervention, we revealed the potential effects of HBD on weight loss and gut microbiota. Our results indicated that the HBD resulted in a significant decrease in body mass index (BMI), and most of the physiological indicators were decreased to a greater degree in the effective HBD group (EHBD, weight loss ≥ 5%) than in the ineffective HBD group (IHBD, weight loss < 5%). The HBD intervention also modified the gut microbiota of the subjects with obesity. Specifically, *Blautia*, *Lachnoclostridium*, *Terrisporobacter*, *Ruminococcus (R. torques*, *R. gnavus)*, and *Pseudomonas* were significantly reduced. In addition, we employed machine learning models, such as XGBRF and GB models, to rank the importance of various features and identified the top 10 key bacterial genera involved. Gut microbiota co-occurrence networks showed the dominance of healthier microbiota following successful weight loss. These results suggested that the HBD intervention enhanced weight loss, which may be related to diet-induced changes in the gut microbiota.

## 1. Introduction

Obesity is a global health crisis that will continue to worsen in the coming years. The last four decades have witnessed a tenfold increase in childhood and adolescent obesity [1]. The World Health Organization has defined obesity as an excessive and potentially harmful fat accumulation. The primary reason behind obesity is the imbalance between the body’s energy intake and expenditure [2]. Not only are people with obesity depressed due to mood and anxiety disorders, but they are at increased risk for several debilitating diseases and health conditions like type 2 diabetes, hypertension, myocardial infarction, stroke, liver cancer, colon cancer, and breast cancer [3].

The current primary treatment strategy to control obesity is to increase the body’s energy expenditure or reduce energy intake. For example, a switch from a sedentary lifestyle to an active lifestyle with the inclusion, where required, of behavioral therapy. The gut microbiota plays a key role in human health by improving digestion and regulating the immune system, and thus this aspect has attracted growing concerns in recent years [4]. There is increasing evidence that imbalances in the gut microbiota may be a crucial factor in obesity [5,6,7,8]. Several studies have indicated that gut-microbiota-derived metabolites play a crucial role in nutrient uptake and energy metabolism of the body, and any irregularities in this process lead to obesity or being underweight [9,10]. Growing evidence suggests that the composition, diversity, metabolic function, and immune function of the gut microbiota in people with obesity are altered compared to the healthy group [11,12]. It has been observed that the composition of gut microbiota in people with obesity differs significantly from that of healthy people. Specifically, there is a notable reduction in beneficial bacteria like *Bifidobacterium* and an increase in potentially pathogenic bacteria such as *Fusobacterium* and *Escherichia-Shigella* [13]. Therefore, a comprehensive gut microbiota analysis can help determine specific microbial signatures associated with health and disease (for example, obesity).

Although the exact mechanisms causing obesity are still obscure, dietary patterns are associated with the onset of obesity and associated comorbidities. For example, although continuous energy restriction (CER) is one of the most common dietary strategies for weight loss and metabolic improvements, long-term adherence to this becomes problematic as it involves restricting energy intake by 15–30% [14]. Intermittent energy restriction (IER) serves as an alternative to CER for weight loss and has been confirmed to yield comparable effects on weight loss and health promotion [15,16]. Furthermore, the characteristics of the gut microbiota are influenced by several factors, including diet. Different dietary interventions can have an impact on the gut microbiota, which includes the application of probiotics and prebiotics. Probiotic intake has been shown to aid anti-obesity interventions by promoting weight loss and reducing cardiovascular risk factors [17]. However, the efficacy of probiotic interventions can be influenced by gut microbiota composition in obese individuals [18]. In obesity treatment, significant progress has been made in diet therapy, with many studies dedicated to exploring ways of controlling obesity through dietary patterns. Several dietary approaches have been extensively studied, including short-term carbohydrate diets [19], high-protein energy-restricted diets [20], Mediterranean diets [21,22], and ketogenic diets [22,23]. As a recommended nutritional pattern, the Mediterranean diet has been found to influence the composition and function of the gut microbiota, contributing to the development of a healthier microbiota [21,22]. On the other hand, the ketogenic diet, characterized by a high-fat, low-carbohydrate formulated diet, has been used to treat epilepsy, diabetes, and obesity and has been shown in clinical studies to have beneficial effects on body weight, metabolic parameters, and gut microbiota composition [22,23].

Different dietary patterns, however, have a variable effect on host metabolism, which is governed by person-specific gut microbiota and body physiology [24]. Due to the individual specificity of the gut microbiota, there may be differences in the response of people with obesity to different or even the same dietary patterns, and we hypothesized that the gut microbiota composition before the intervention could influence the metabolic and microbial responses produced by the HBD intervention in people with obesity. To verify this hypothesis, we designed a 12-week HBD intervention to assist with weight loss on top of fundamental exercises for people with obesity. A series of analyses were performed on the gut microbiota before and after HBD intervention, including alpha diversity, beta diversity, linear discriminant analysis effect size (LEfSe), and co-occurrence network analyses. Thus, this work indicated the role of the gut microbiota profile of subjects with obesity in determining the efficacy of HBD in regulating obesity and disorders thereof. This knowledge provides a clinical basis for the development of effective dietary intervention strategies based on the obese-affected gut microbiota profile. In addition, to further identify potential biomarkers of people with obesity, machine learning (ML) algorithms were utilized to classify people with obesity before and after receiving balanced diet interventions. Using feature importance information from classifiers to determine which bacteria are most associated with obesity is an effective research approach.

## 2. Materials and Methods

### 2.1. Materials and Diet

The trial was conducted in the Affiliated Wuxi People’s Hospital of Nanjing Medical University (Wuxi, China) and ratified by the hospital’s ethics committee (KYLLKS 201806). The recruitment of subjects began after registering at the Chinese Trial Registry (ChiCTR1800015923). The inclusion criteria were as follows: age of 18–65 years, body mass index (BMI) ≥ 28 kg/m^2^, and stable body weight (BW) during the first three months. Volunteers were excluded if they accorded with any specific criteria in the previous analysis [25]. 

All subjects underwent a 4-week run-in period to guide diet, aerobic, and resistance exercises, followed by a 12-week intervention phase aiming to lose weight. The nutrient composition of the HBD was 24% protein, 37% fat, and 39% carbohydrate, as determined according to the participant’s basal metabolic rate. The basal metabolic rate measured using the InBody S10 body bioelectrical impedance analyzer was multiplied by 1.2 to calibrate the total calories needed (Table 1).

For vegetables, subjects could choose tender stems, leaves, cauliflower, onion and garlic, fungus and algae, eggplant, and aquatic vegetables. For lean meat, subjects could choose lean livestock and poultry meat. Aquatic products could include freshwater and saltwater fish, freshwater and saltwater shrimp, and shellfish. Subjects could also eat fruits (i.e., apple, kiwi, and citrus); nuts such as almonds and cashews; and oil, including olive oil, linseed oil, and tea seed oil.

The exercise scheme designed for the HBD subjects included aerobic and resistance exercises, and the specific plan was described in the previous analysis [25]. 

Before study initiation, nutritionists informed the subjects of the weight loss plan and exercise regimen. They also closely communicated with the subjects and provided guidance throughout the study. The subjects reported their diet, food weight, body weight, and any physical discomfort through pictures and text in a WeChat group daily. They participated in the appropriate aerobic and resistance exercise regimen while receiving the dietary intervention. During the implementation of the program, the nutritionists followed up with the patients on WeChat daily. In the case of problems, the nutritionists discussed and proposed solutions in addition to maintaining good records and conducting monthly outpatient consultations.

### 2.2. Anthropometric Evaluation

BW, height, systolic and diastolic blood pressure (SBP and DBP), waist circumference (WC), and hip circumference (HC) were measured at 0 and 12 weeks. All measurements of paraments were obtained in the previous analysis.

### 2.3. Blood Chemistry Paraments Determination

Concentrations of albumin, fasting blood glucose [FBG], creatinine, triglyceride [TG], total cholesterol [TC], high-density lipoprotein cholesterol [HDL-c], low-density lipoprotein cholesterol [LDL-c], blood urea nitrogen [BUN], uric acid, alanine transaminase [ALT], aspartate transaminase [AST], gamma-glutamyl transpeptidase [GGT], and alkaline phosphatase [ALP] were measured after an 8 h fast at 0 and 12 weeks using the AU5800 clinical chemistry analyzer (Beckman Coulter, Inc., Sykesville, MD, USA). The hemoglobin A1c level [HbA1c] was also measured before and after the HBD using VARIANT II Hemoglobin Testing System (Bio-Rad, Hercules, CA, USA).

### 2.4. Fecal Samples Collection and Storage

As the target of this study was to investigate the effects of changes in the gut microbiota of subjects with obesity before and after the HBD, fecal samples were collected in collection tubes from the study subjects at 0 and 12 weeks for subsequent bacterial composition determination and amplicon sequencing. Briefly, the subjects were asked to empty their bowels into a clean container to prevent sample contamination. After the fecal sample was collected, the collection date was marked on the collection tube, and the sample was immediately stored at −80 °C to stabilize its microbial composition. In addition, fecal samples were transported on dry ice in the dark.

### 2.5. DNA Extraction from Fecal Samples

DNA extraction was performed for each fecal sample according to the manufacturer’s instructions, using the FastDNA Spin Kit for Feces (MP Biomedicals, Solon, OH, USA). The 16 S rRNA V3–V4 hypervariable region was amplified using the 16 S V3 314 F for-ward and V4 806 R reverse primers. Subsequent sequencing using specific adapters was performed on the Illumina MiSeq PE300 platform (Illumina, San Diego, CA, USA). The resulting amplification solution was purified with a Gel/PCR Extraction Kit (Biomiga Inc., San Diego, CA, USA). DNA libraries were prepared using the TruSeq DNA LT Sample Preparation Kit (Illumina).

The fecal samples were pretreated before DNA extraction. The fecal samples (500 mg) without glycerin were directly added to the Lysing Matrix E tube. The fecal samples containing glycerol were centrifugated at 13,000 g for 3 min after thawing, then the supernatant was removed, and 500 mg residue was added to the Lysing Matrix E tube.

### 2.6. Bioinformatics Analysis 

#### 2.6.1. Gut Microbiota Analyses

The previous article explained the process for obtaining the OTU table using qiime2-2022.2 software to analyze the gut microbiota data [25]. Subsequently, the OTU table was extracted and normalized to generate the relative abundance tables at both the phylum and genus levels.

In addition, we employed various indices to assess alpha diversity, including the phylogenetic diversity (PD) index, the Shannon index, the Pielou evenness index, and the observed OTU counts. To analyze alpha diversity, we utilized R packages such as ggplot2 [26] and ggsignif [27] for data visualization and significance annotation.

We utilized the vegan package for beta diversity analysis to calculate Bray–Curtis phase dissimilarity and unweighted and weighted UniFrac distances. We then explored the resulting phase dissimilarity matrix using multivariate techniques from R packages such as ade4 [28] and psych [29]. In summary, we utilized R packages ggplot2 [26] and ggsignif [27] to visualize alpha diversity, while R packages vegan [30], ade4 [28], and psych [29] were employed to investigate patterns of beta diversity. These R packages provide crucial tools for analyzing and visualizing the diversity of microbial communities in our study. LEfSe [31] was exploited to recognize the bacterial taxa leading to differences before and after the HBD intervention. To construct the co-occurrence network, the genera with relative abundances of less than 0.1% were removed at first. Secondly, the Spearman correlation coefficients of each genus in a single group were calculated by using the corr. test function in R package psych [29], and the correlation coefficient matrix and *p*-value matrix were obtained. The Benjamini–Hochberg False Discovery Rate (FDR) method was used to correct the *p*-value obtained in the above steps [32]. Lastly, the co-occurrence network of gut microbiota of two groups was established based on the Spearman correlation matrix and corrected *p*-value matrix [33]. The co-occurrence networks were constructed in the condition of correlation coefficients (r > |0.6|) and the false discovery rate (*p* < 0.05) and were visualized using Gephi 0.9.6 software [34].

#### 2.6.2. Construction of Machine Learning Binary Classifiers

This study employed machine learning models to assess classification accuracy and identify key bacterial genera. To model the relative abundance of bacterial genera before and after the intervention, we employed eight models, namely k-nearest neighbor (kNN), support vector machine (SVM), decision tree (DT), random forest (RF), gradient boosting regression tree (GB), extreme gradient boosting (XGB and XGBRF), and LightGBM (LGB), to predict classification effectiveness. To reduce model overfitting and provide a more accurate performance evaluation, we utilized 10-fold cross-validation with 5 repetitions. The area under the ROC curve (AUC) was chosen as the model performance evaluation index. Furthermore, in descending order, we extracted the top 10 feature importance based on the feature importance of DT, RF, GB, XGB, XGBRF, and LGB. Among these models, KNN, SVM, DT, RF, and GB were constructed using the scikit-learn package [35]. XGB and XGBRF were built using the XGBoost package [36], while LGB was implemented using the Lightgbm package [37].

### 2.7. Statistical Analyses 

Statistical analyses were conducted using the R Statistical Package (version 4.2.0; https://www.r-project.org/ (accessed on 22 April 2022)). Comparisons of anthropometric assessment indicators before and after the HBD were conducted using the Wilcoxon rank-sum test or paired *t*-test. The data are presented as the mean ± standard deviation. The Shapiro–Wilk test was used to determine whether the data were normally distributed. To compare the data, we used the paired *t*-test for normally distributed data and the Wilcoxon rank-sum test for non-normally distributed data. We compared the gut microbiota diversity abundance in the phylum and genus taxon between the two groups before and after the HBD using the Wilcoxon rank-sum test in the R statistical package. A *p*-value < 0.05 was considered statistically significant.

## 3. Results

### 3.1. Differential Response to HBD Based on the Anthropometric and Clinical Blood Chemistry Parameters in Subjects with Obesity

Forty-three subjects (72% male and 28% female), with a mean age of 33.60 ± 8.44 years, a mean weight of 88.54 ± 13.33 kg, and a mean height of 168.33 ± 6.64 cm, were included in this study. The subjects were not significantly different (based on the Wilcoxon rank-sum test or paired *t*-test) in terms of their age, weight, height, and BMI. At week 0, the mean BMI of the subjects was 31.13 ± 3.22 kg/m^2^. At week 12, there was no statistically significant change in weight loss among the subjects, whereas the mean weight loss was 4.1%. In addition, after the intervention, levels of weight, BMI, WC, HC, uric acid, LDL-c, and TG decreased, but not statistically (Table 2). However, only WC, creatinine, and FBG significantly reduced (*p* < 0.05; Table 2). These improvements in anthropometric and blood biochemical indicators essentially indicated an improvement in obesity status. The weight loss success rate among the subjects was 44% (weight loss ≥ 5% was regarded as weight loss success). The subjects were then divided into two groups based on successful weight loss: those with weight loss below 5% comprised the ineffective HBD group (IHBD), and those with weight loss not less than 5% comprised the effective HBD group (EHBD).

(1) $$\mathrm{weight}\;\mathrm{loss}=\frac{\mathrm{BW}1-\mathrm{BW}2}{\mathrm{BW}1}\times 100\%$$

BW1: BW was measured before the intervention; BW2: was measured after the intervention.

**Table 2:** Subjects’ anthropometric and clinical blood chemistry parameters before and after the HBD intervention.

Index	Pre-HBD	Post-HBD	*p*	Change	*n*
BW [kg]	88.5 ± 13.3	85.0 ± 13.8	0.283	−3.5 ± 3.3	43
BMI [kg/m^2^]	31.1 ± 3.2	29.9 ± 3.5	0.087	−1.3 ± 1.1	43
WC [cm]	100.8 ± 9.3	94.4 ± 17.8	0.026	−4.4 ± 3.2	43
HC [cm]	105.2 ± 6.5	101.1 ± 17.3	0.293	−1.9 ± 2.3	43
SBP [mmHg]	129.2 ± 11.6	126.3 ± 13.3	0.082	−2.9 ± 10.6	43
DBP [mmHg]	74.7 ± 10.3	72.7 ± 9.6	0.188	−2.0 ± 9.9	43
Albumin [g/L]	46.1 ± 2.4	45.7 ± 2.7	0.377	−0.3 ± 2.4	41
ALT [U/L]	46.0 ± 29.5	31.7 ± 18.4	0.117	−14.3 ± 19.9	43
AST [U/L]	28.4 ± 11.4	25.1 ± 20.9	0.066	−3.3 ± 22.4	43
ALP [U/L]	89.8 ± 22.3	85.6 ± 18.2	0.051	−4.2 ± 13.6	43
GGT [U/L]	37.6 ± 24.4	33.3 ± 19.8	0.616	−4.3 ± 13.5	43
BUN [mmol/L]	5.1 ± 1.4	5.3 ± 1.5	0.952	0.3 ± 1.2	43
Creatinine [µmol/L]	76.0 ± 13.3	74.2 ± 12.6	0.030	−1.8 ± 5.3	43
Uric acid [µmol/L]	429.2 ± 104.4	412.9 ± 99.3	0.061	−16.3 ± 55.5	43
TG [mmol/L]	2.0 ± 1.2	1.9 ± 1.1	0.378	−0.1 ± 0.9	43
TC [mmol/L]	4.8 ± 0.9	4.9 ± 0.9	0.509	0.1 ± 0.6	43
LDL-c [mmol/L]	3.0 ± 0.9	2.9 ± 0.8	0.634	−0.1 ± 0.5	43
HDL-c [mmol/L]	0.9 ± 0.2	1.0 ± 0.2	0.102	0.1 ± 0.1	43
FBG [mmol/L]	5.2 ± 0.6	5.0 ± 0.5	0.005	−0.2 ± 0.5	43
HbA1c [%]	5.1 ± 0.4	5.2 ± 0.4	0.138	0.1 ± 0.4	43

Abbreviations: BW, body weight; BMI, body mass index; WC, waist circumference; HC, hip circumference; SBP, systolic blood pressure; DBP, diastolic blood pressure; ALT, alanine transferase; AST, aspartate transaminase; ALP, alkaline phosphatase; GGT, gamma-glutamyl transpeptidase; BUN, blood urea nitrogen; TG, triglyceride; TC, total cholesterol; LDL-c, low-density lipoprotein cholesterol; HDL-c, high-density lipoprotein cholesterol; FBG, fasting blood glucose; HbA1c, hemoglobin Alc. The *p*-value is calculated using the Wilcoxon rank-sum test or paired *t*-test and represents the difference before and after HBD intervention. The *n-*number is the number of samples before and after the HBD intervention.

The statistical analysis results of the changes in anthropometric and blood biochemical indicators during the study in the EHBD and IHBD groups are shown in Table 3 and Table 4, respectively. The levels of weight, BMI, WC, SBP, Albumin, ALP, Creatinine, LDL-c, and FBG decreased significantly in the EHBD group after the intervention, while the levels of HDL-c increased significantly (Table 3). Of the anthropometric and blood chemistry parameters, only weight, HDL-c, and AST changed significantly after the intervention in the IHBD group (Table 4). In addition, we noted a downward but not significant trend in HC, DBP, ALT, GGT, uric acid, TG, and TC in the EHBD group (Table 3). Moreover, at the end of the 12-week intervention, consistent changes in weight, BMI, WC, HC, SBP, DBP, ALT, GGT, BUN, creatinine, uric acid, HDL-c, and FBG were observed in both the EHBD and IHBD groups. These results indicated that the HBD intervention improves the anthropometric and blood chemistry parameters of subjects with obesity. In summary, the implementation of HBD, especially in the EHBD group, has shown positive outcomes in influencing the health of individuals with obesity by enhancing various anthropometric and blood chemistry parameters.

### 3.2. HBD Alters the Gut Microbiota in Subjects with Obesity 

**Figure 1:**
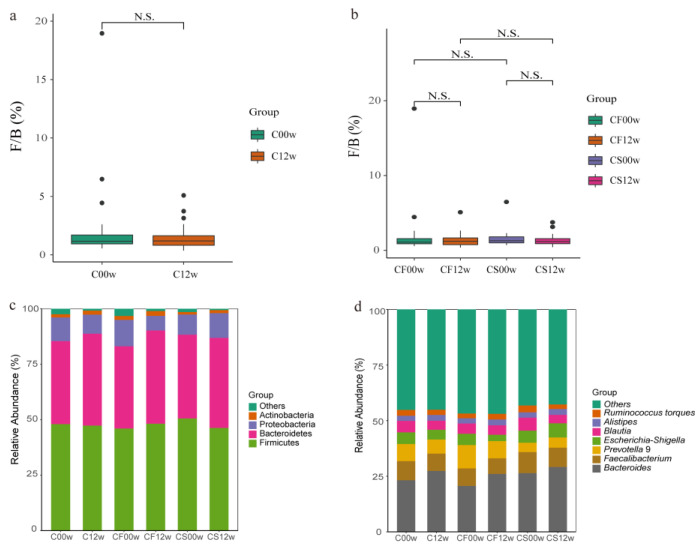
Differences in the gut microbiota before and after the HBD in the EHBD and IHBD groups. (**a**) The ratio of Firmicutes to Bacteroidetes at the phylum level (N.S.: *p* > 0.05). C00w: before the HBD intervention; C12w: after the intervention. Differences in the gut microbiota before and after the intervention in the EHBD and IHBD groups; (**b**) the ratio of Firmicutes to Bacteroidetes at the genus level (N.S.: *p* > 0.05); (**c**) gut microbiota composition at the phylum level; (**d**) gut microbiota composition at the genus level.

**Figure 2:**
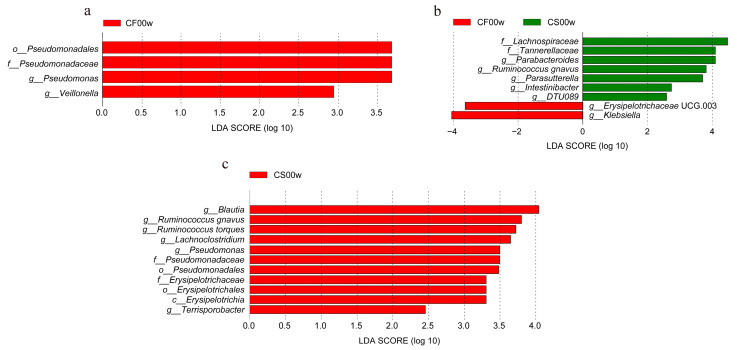
Differences in the gut microbiota before and after the HBD in the EHBD and IHBD groups. (**a**–**c**) LEfSe plot showing the unique genus signatures identified. CF00w: before the intervention, IHBD; CF12w: after the intervention, IHBD; CS00w: before the intervention, EHBD; CS12w: after the intervention, EHBD.

Differential abundance of OTUs at the phylum, class, order, family, and genus levels of bacterial taxonomic classification revealed differences in the relative abundance of several taxa before and after the HBD intervention (Figure 1a–d and Figure 2a–c). At the phylum level, the predominant phyla before and after the HBD were Firmicutes, Bacteroidetes, Proteobacteria, and Actinobacteria (Figure 1c). At the class level, *Erysipelotrichia* was significantly reduced (*p* < 0.05) in the EHBD group after 12 weeks of treatment, whereas no significant changes were observed in the IHBD group (Figure 2a,c). At the order level, a dramatic decrease in *Pseudomonadales* and *Erysipelotrichales* was observed after the intervention (*p* < 0.05) in the EHBD group, and *Pseudomonadales* also decreased in the IHBD group (Figure 2a–c). At the family level, the EHBD group showed a dramatic decrease in *Pseudomonadaceae* and *Erysipelotrichaceae*, while *Pseudomonadaceae* decreased after the intervention (Figure 2c). At the genus level, the predominant groups before and after HBD included *Bacteroides*, *Faecalibacterium*, *Prevotella* 9, *Escherichia-Shigella*, *Blautia*, *Alistipes*, and *Ruminococcus torques* (Figure 1d). Meanwhile, LEfSe analysis revealed a significant reduction in some genera after HBD intervention (Figure 2a–c); for example, *Blautia*, *Lachnoclostridium*, *Ruminococcus (R. torques*, *R. gnavus)*, and *Terrisporobacter* were dramatically reduced in the EHBD group (*p* < 0.05). The significant reduction in *Veillonella* (*p* < 0.05) was unique to the IHBD group. After the HBD intervention, *Pseudomonas* significantly reduced (*p* < 0.05) in the gut microbiota of the EHBD and IHBD groups. Comparing the characteristic bacterial genera of the baseline gut microbiota between the EHBD and IHBD groups, we discovered that the EHBD group had more characteristic bacterial genera at baseline (at the beginning of the trial). For example, *Parabacteroides*, *Ruminococcus gnavus*, *Parasutterella*, *Intestinibacter*, and DTU089 were characteristic genera in the EHBD group, whereas *Erysipelotrichaceae* UCG 003 and *Klebsiella* were characteristic genera in the IHBD group (Figure 2d).

**Figure 3:**
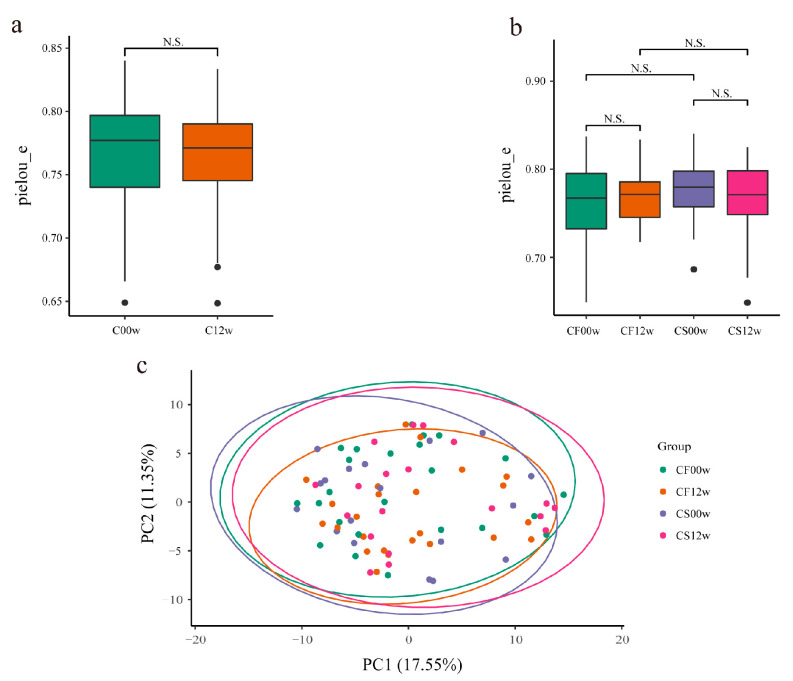
Differences in the gut microbiota before and after the HBD. (**a**) Alpha diversity based on the pielou_e index (N.S.: *p* > 0.05); (**b**) alpha diversity based on the pielou_e index (N.S: *p* > 0.05); (**c**) beta diversity. CF00w: before the intervention, IHBD; CF12w: after the intervention, IHBD; CS00w: before the intervention, EHBD; CS12w: after the intervention, EHBD.

Faith’s phylogenetic diversity (PD) index, the Shannon index, Pielou’s evenness index, and observed OTUs were calculated to gain insights into changes in the gut microbiota before and after the HBD intervention (Figure 1a,b and Supplementary Material Figure S1a–f). These indices showed the richness and diversity of the gut microbiota and the evenness of the distribution of microbial community abundances. There were no striking differences in Faith’s PD index, the Shannon index, Pielou’s evenness index, and observed OTUs in the microbiota before and after the HBD intervention (Figure 3a and Supplementary Material Figure S1a–c). However, there was a slight increase in Pielou’s evenness index in the IHBD group and a slight decrease in the EHBD group (Figure 3b). This indicated that after the HBD intervention, bacterial genera uniformity of the gut microbiota increased in the IHBD group and decreased in the EHBD group. In addition, Faith’s PD index, the observed OTUs, and the Shannon index in the EHBD and IHBD groups were decreased (Supplementary Material Figure S1d–f), indicating that the OTU complexity and community diversity of the gut microbiota decreased in both groups. 

Beta diversity reflects the variation in bacterial genera abundance distribution among samples. The beta diversity in both HBD groups was calculated using the Bray–Curtis dissimilarity, weighted, and unweighted UniFrac analyses (Supplementary Material Figure S2a–f). Beta diversity in the gut microbiota did not differ significantly before and after the intervention in the EHBD and IHBD groups (Figure 3c), which indicated that the structure of the gut microbiota after the intervention was highly similar to that before the intervention. Based on the Wilcoxon rank-sum test, the EHBD and IHBD groups showed significant differences in the Bray–Curtis dissimilarity and unweighted and weighted UniFrac distances (Supplementary Material Figure S2d–f). It is worth noting that there were significant differences between CS00w and CF00w (before the intervention in the EHBD and IHBD groups, respectively) based on the Bray–Curtis dissimilarity and weighted UniFrac distances. In addition, based on the Bray–Curtis dissimilarity, the gut microbiota structure of the CS12w group (after the intervention in the EHBD group) was found to be dramatically different from those in the CF12w group (after the intervention in the IHBD group) after the HBD intervention. Furthermore, the difference in the EHBD group was more dramatic than that in the IHBD group before and after the intervention (Supplementary Material Figure S2d). Based on the weighted UniFrac distance, the gut microbiota structure of the subjects in the CS12w and CF12w groups was more similar to each other after the intervention and significantly different from the gut microbiota structure of the subjects before the intervention (Supplementary Material Figure S2c). This showed that the gut microbiota structure of the subjects changed after the HBD intervention, which was more evident in the EHBD group. More importantly, differences in the gut microbiota at baseline between the EHBD and the IHBD groups may be for the point which resulted in successful weight loss in only one of the groups.

In summary, the significantly altered genera in the EHBD group may serve as key reference indicators for dietary intervention in the gut microbiota of subjects with obesity.

### 3.3. Machine Learning Models Reveal the Importance of Gut Microbiota Characteristics in Subjects with Obesity

**Figure 4:**
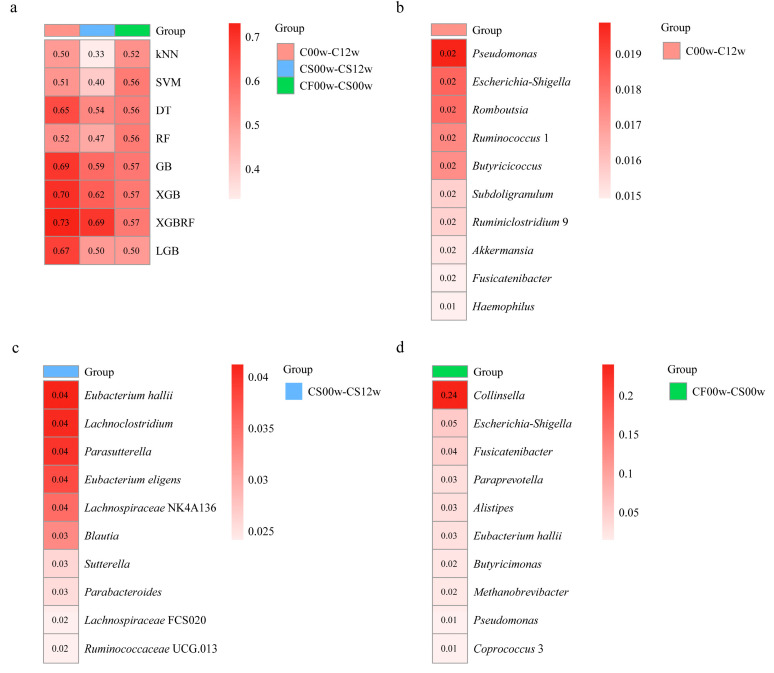
Performance of a binary classification model based on bacterial genera composition of the gut microbiota. (**a**) The ability of eight machine algorithms to predict the bacterial genera composition (evaluation metric is AUC); (**b**) top 10 key bacterial genera for subjects with obesity based on the XGBRF model before and after the HBD intervention; (**c**) top 10 key bacterial genera for subjects with obesity based on XGBRF model before and after the EHBD intervention; (**d**) top 10 key bacterial genera for subjects with obesity based on the GB model before the EHBD and IHBD intervention.

Based on the dataset before and after the HBD intervention, a machine learning binary classifier was developed to classify the samples accurately. According to the model evaluation, the XGBRF model had the best classification performance with an AUC value of 0.73 (Figure 4a). Therefore, the XGBRF model was chosen to determine the feature importance in the subjects with obesity. The results showed that *Pseudomonas*, *Escherichia-Shigella*, *Romboutsia*, *Ruminococcus* 1, *Butyricicoccus*, *Subdoligranulum*, *Ruminiclostridium* 9, *Akkermansia*, *Fusicatenibacter*, and *Haemophilus* were the top 10 key bacterial genera for the subjects with obesity (Figure 4b). Before and after the EHBD intervention, the model with the best classification performance according to model evaluation was also XGBRF with an AUC value of 0.69 (Figure 4a). The results showed that the top 10 key bacterial genera in terms of feature importance included *Eubacterium hallii*, *Lachnoclostridium*, *Parasutterella*, *Eubacterium eligens*, *Lachnospiraceae* NK4A136, *Blautia*, *Sutterella*, *Parabacteroides*, *Lachnospiraceae* FCS020, and *Ruminococcaceae* UCG 013 (Figure 4c). Before the EHBD and IHBD intervention, the model with the best classification performance according to model evaluation was GB, with an AUC value was 0.57 (Figure 4a). The top 10 key bacterial genera in order of characteristic importance score were obtained as follows: *Collinsella*, *Escherichia-Shigella*, *Fusicatenibacter*, *Paraprevotella*, *Alistipes*, *Eubacterium hallii*, *Butyricimonas*, *Methanobrevibacter*, *Pseudomonas*, and *Coprococcus* 3 (Figure 4d).

Among the above-listed genera, *Pseudomonas* has the most critical role as a key bacterial genus before and after HBD. Although *Pseudomonas* was not among the top 10 feature importance of the EHBD group, it still belonged to the key genera when considering the Lefse results (Supplementary Material Figure S3a). In addition, in a previous study, pre-intervention levels of *Butyricoccus* promoted a response to inulin, leading to a decrease in body mass index in subjects with obesity [18]. Based on our findings, it was found that *Butyricoccus* was able to distinguish to some extent between subjects who had received the HBD intervention and those who had not. The box plot clearly showed that the abundance of this genus in the gut of subjects with obesity increased after the HBD intervention (Supplementary Material Figure S3b). This finding suggested that *Butyricoccus* responded positively to a balanced diet as an intervention. Surprisingly, in our study, *Blautia* showed a decline after the HBD intervention, regardless of whether the subjects had positive responses in their gut microbiota (Supplementary Material Figure S3c). However, many studies have shown that *Blautia* was negatively correlated with the visceral fat area at the genus level [18,38]. In the EHBD group, in addition to a significant reduction in *Blautia* after the intervention, there was also a significant reduction in *Lachnoclostridium* (Supplementary Material Figure S3d).

In summary, machine learning methods provided valuable tools and opportunities for a deeper understanding of the complex issue of obesity. Through model evaluation and feature importance analysis, key features of obesity can be identified, enabling a more profound comprehension of the underlying mechanisms. Furthermore, this knowledge can aid in developing effective strategies to combat obesity.

### 3.4. HBD Alters the Gut Microbiota Co-Occurrence Networks in Subjects with Obesity 

To investigate the differences in the bacterial interaction patterns of gut microbiota before and after HBD, we constructed co-occurrence networks filtered with the following criteria: strong correlation (|r| > 0.6) and *p*-value < 0.05. A total of 152 positive and 16 negative significant correlations were found between 114 genera in the EHBD group before the HBD (Table 5), compared with 133 positive and 21 negative significant correlations between 111 genera after the HBD intervention (Table 5). In addition, 78 positive and six negative significant correlations were found between 111 genera in the IHBD group before the HBD (Table 6), compared with 77 positive and nine negative significant correlations between 108 genera after the HBD intervention (Table 6).

**Figure 5:**
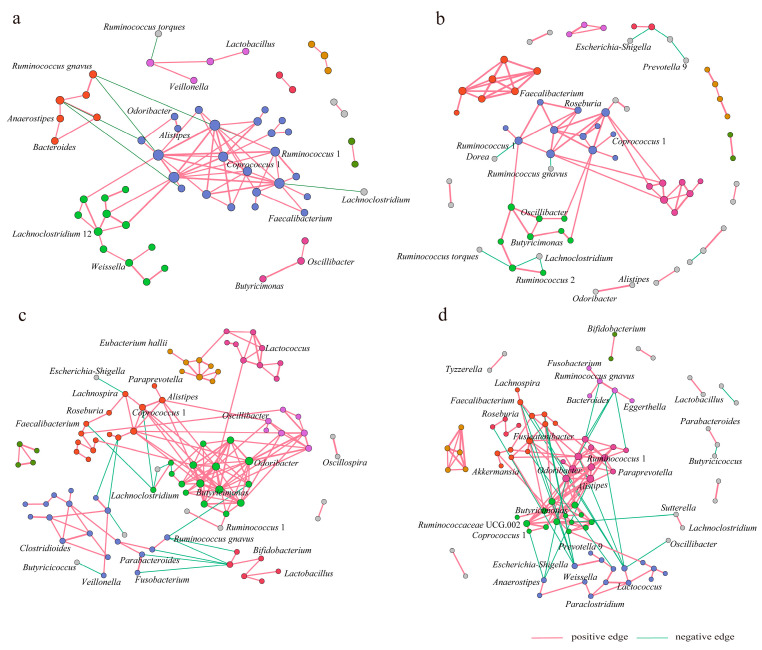
The HBD alters the gut microbiota co-occurrence network. The main network nodes are labeled by the genus, and the interactions between genera are composed of nodes and edges. (**a**) CF0 network; (**b**) CF12 network; (**c**) CS0 network; (**d**) CS12 network. CF0: before the intervention, IHBD group; CF12w: after the intervention, IHBD; CS00w: before the intervention, EHBD; CS12w: after the intervention, EHBD.

After the HBD intervention, the average clustering coefficients increased from 0.258 to 0.484 in the gut microbiota co-occurrence networks in the IHBD group, whereas the clustering coefficients generally decreased in the EHBD group, indicating individual differences in the complexity of co-occurrence networks. We applied a modularity algorithm in Gephi to cluster bacterial nodes into subgroups with a higher connection density between nodes in subgroups than in non-subgroup networks. There were two major subclusters with more than 10 nodes recognized in the CF0 network and one major subcluster with more than 10 nodes identified in the CF12 network (Figure 5a,b), while the number of main subclusters with >10 nodes increased from 2 to 4 in the CS0 and CS12 co-occurrence networks (Figure 5c,d).

After the HBD intervention, the nodes and interactions between genera in gut microbiota co-occurrence networks changed in the EHBD and IHBD groups. *Ruminococcus* 1 and *Lachnoclostridium* 12 dominated two major modules of the CF0 network, respectively, while *Ruminococcus* 1 dominated one major module of the CF12 network. *Alistipes*, *Odoribacter*, and *Butyricimonas* dominated two major modules of the CS0 network, while *Fusicatenibacter*, *Ruminococcus* 1, *Lactococcus*, and *Ruminococcaceae* UCG 002 dominated in the four major modules of the CS12 network. Specifically, HBD altered the interactions of the core genera associated with obesity in the gut microbiota network of EHBD group patients; for example, the interactions of *Escherichia-Shigella*, *Faecalibacterium*, and *Alistipes* with other genera increased. Before the intervention, the pathogenic bacterial genera *Escherichia-Shigella* had a negative association with *Lachnospira*. However, this genus showed positive associations with *Faecalibacterium*, *Fusicatenibacter*, *Lachnospiraceae* UCG 001, *Ruminococcus gauvreauii*, and *Lachnospiraceae* NK4A136 after the intervention. In addition, HBD altered the interactions of the non-core genera connected with obesity in the gut microbiota network of the EHBD group. The interactions of *Ruminococcus* 1, *Weissella*, and *Akkermansia* with other genera were found to be increased. However, the interactions of *Roseburia*, *Lactobacillus*, *Bifidobacterium*, *Lachnospira*, *Odoribacter*, and *Butyricicoccus* with other genera decreased. In the IHBD group, the interaction between *Dorea* and *Ruminococcus* 1 and the interactions between *Butyricicoccus* and other genera enhanced, but the interactions of *Ruminococcus gnavus*, *Coprococcus* 1, and *Weissia* with other genera became weak. 

In summary, after the HBD, subjects in the EHBD group lost weight successfully. Their gut microbiota co-occurrence networks tended to be normal, but the interaction relationships between some conditioned pathogenic bacterial genera and other bacterial genera were enhanced.

## 4. Discussion

Diet is recognized as a vital factor affecting the diversity and function of the gut microbiota. However, high-calorie diets and food containing high-fat content are not considered healthy. The regular intake of such diets results in the disruption of energy balance, leading to several metabolic disorders, such as obesity. Moreover, high-calorie and high-fat diets alter the gut microbiota diversity, leading to the loss of gut barrier function [39]. However, a recent study confirmed that caloric restriction helps reduce weight loss and improve the gut microbiota profile and associated functions [40,41,42]. Therefore, nutritional intervention with HBD affects the gut microbiota composition of subjects with obesity, making those genera dominant, which regulate human metabolism.

It is equally important to determine the taxonomic changes induced by the HBD intake so that role of the specific gut microbiota in relation to obesity is highlighted. In this study, LEfSe analysis showed some genera that were significantly reduced in the EHBD group, including *Blautia*, *Lachnoclostridium*, *Ruminococcus* (*R. torques*, *R. gnavus*), and *Terrisporobacter.* Among them, *Blautia and Lachnoclostridium* were also the top 10 key genera of the EHBD group before and after the intervention. Therefore, we speculated that the decrease in abundance of these genera may be responsible for 5% of the weight loss. Previously, the abundance of *Blautia* was confirmed to be significantly positively associated with BMI [43]. *Blautia* composition has been reported to be associated with eating patterns and influenced by dietary fat content. A cross-sectional study in human adults confirmed the inverse relationship between *Blautia wexlerae* and obesity and further confirmed its ameliorative effects on obesity via metabolic remodeling of the gut microbiota [44]. Another cross-sectional study on the compositional changes in the gut microbiota with aging showed a reduction in the overall gut microbiota diversity with age, including a significant reduction in the relative abundance of *Blautia* [45]. Our results on the inverse association of other bacterial genera with obesity also align with some previous findings. A correlation study between the gut microbiota and obesity-related indicators in human subjects showed a significantly positive association between *Lachnoclostridium* and BW [46]. Although *Ruminococcus gnavus* was initially identified in 1974 as a strictly anaerobic bacterium in the healthy human gut [47], some studies have indicated its overrepresentation in certain diseases [48], such as obesity and other metabolic diseases. Moreover, it has been observed to decrease in abundance during weight loss, consistent with earlier studies [49]. In addition, it has been shown that *Ruminococcus gnavus* is an opportunistic pathogen and is positively associated with obesity [50]. Another study reported the association of *Ruminococcus torques* and *Ruminococcus gnavus* with visceral fat accumulation [51]. Therefore, it is speculated that a reduction in visceral fat due to successful weight loss leads to a reduction in these two genera. In the IHBD group in the current study, *Veillonella* was the only genus that showed a decrease in relative abundance, and that made the two groups (IHBD and EHBD) different. *Veillonella* can metabolize lactic acid and reduce the insulin resistance induced by lactic acid in subjects with obesity [52], so it can be inferred that although weight loss does not reach 5% in some patients, the gut microbiota may change beneficially. There have been few reports on the association between *Pseudomonas* and obesity. Our results showed that *Pseudomonas* decreased with weight loss in both the EHBD and IHBD groups, suggesting that the relationship between *Pseudomonas* and an HBD is worth further study. 

There are multiple microbial communities in the human gut, in which the organisms interact closely with each other. However, our understanding of the role of the gut microbiota in a diet influencing obesity depends mainly on the difference in microbial abundance, whereas little is known about the role of microbial interactions in obesity [53]. In the current study, we utilized co-occurrence network analysis to find associations in bacterial genera based on 16S rRNA sequencing. The key network bacterial genera identified in subjects with obesity highlight their potential roles in modulating the microbial ecosystem in the context of obesity. Our study demonstrated that the co-occurrence networks in the gut microbiota were significantly altered after 12 weeks of the HBD intervention. In the EHBD group, the reduction in network complexity and modularity suggested that the symbiotic network of the gut microbiota appeared to have become simpler after the HBD intervention, but this was not found in the IHBD group. The abundance of *Escherichia-Shigella* has been found to significantly increase in subjects with obesity [13,54]. *Escherichia-Shigella* and *Klebsiella*, as genera of potentially pathogenic bacteria, were found to be positively correlated with each other in the CS12 network, suggesting that there may be synergistic effects between harmful bacteria. Significant differences in dominant genera between the CF0 and CS0 networks suggested that successful weight loss may be related to the gut microbiota composition at baseline. In addition, a study validated our hypothesis that differences in baseline microbiota can predict weight loss on calorie-restricted diets [46]. 

According to our results, there is some discrepancy between the key features of obesity identified by machine learning models and the genus of features obtained using Lefse analysis. This discrepancy may be due to the different methods and algorithms used and the complexity of the obesity problem. Therefore, we focused our study on those genera identified as key genera by multiple methods to gain insight into their impact on obesity. By combining multiple methods and results, we can more fully understand the characteristics and influencing factors of obesity.

However, an intriguing finding emerged from our results, revealing no significant differences in alpha diversity between the groups at baseline and no observed changes over time after the HBD intervention. This indicated that the diet intervention had little effect on the uniformity and richness of the gut microbiota in people with obesity; however, significant differences in the overall structure of the microbiota were still observed through beta diversity analysis. Additionally, various analytical methods, including LEfSe analysis and machine learning models, allowed us to identify key bacterial genera in the gut microbiota of obese individuals before and after the HBD intervention.

Overall, these results indicated that co-occurrence networks in the gut microbiota of subjects with obesity are reorganized into fitter states after successful weight loss. More importantly, our study identified several crucial genera interactions after the HBD intervention in subjects with obesity that may modulate microbial ecosystems. In brief, microbial interactions can promote the implementation of precise diets in clinical practice based on gut microbiota to control obesity. However, there are some things that could be improved in this research. A longer intervention time is needed to assess the effectiveness of the HBD intervention. There is a need to investigate more deeply the potential causal relationship between the efficacy of HBD interventions in subjects with obesity and in which the changes in specific bacteria were observed for the gut microbiota. It is important to note that our study focused on bacterial identification at the genus level. In the future, more state-of-the-art techniques like metagenomics and metabolomics will be employed to comprehensively understand gut microbiota changes in response to the HBD intervention. The aforementioned approaches will help us to accurately predict potential targets and key bacterial genera that respond towards HBD, which would help to develop strategies to intervene in obesity.

## 5. Conclusions

To summarize, our study demonstrated that the HBD intervention enhanced weight loss, which has the potential to connect with diet-induced alterations in the gut microbiota. Notably, only the subjects in the EHBD group exhibited a positive response to the intervention, and the significantly altered genera in this group could serve as key indicators for dietary intervention, targeting the gut microbiota in subjects with obesity. Moreover, the gut microbiota dramatically differed from before to after the HBD intervention concerning its co-occurrence networks, which was reflected by the more significant reduction in the degree of network interaction in the EHBD group compared with the IHBD group after the HBD. In summary, the gut microbiota co-occurrence networks of the study subjects were restructured to a healthier state after weight loss. We have emphasized the features and variations at baseline in the gut microbiota between the EHBD and IHBD groups, which can serve as a reference for predicting the likelihood of successful weight loss in individuals with obesity. In addition, we can determine the contribution of machine learning classification binary models to the characteristic bacterial genera of the gut microbiota by the results of the characteristic importance ranking and further identify the most crucial characteristic bacteria, which can help to gain insight into the relationship between gut microbiota composition and obesity and dietary intervention strategies. Therefore, significant changes in the bacterial abundance and interactions of the gut microbiota may be considered key indicators for dietary intervention in subjects with obesity. All in all, our results highlighted the effects of microbiota interaction on diet-induced changes in the gut microbiota to more fully understand the mechanistic association between diet and host pathologies such as obesity.

## Figures and Tables

**Table 1:** Nutrient composition of the HBD.

Nutrient Composition	HBD ^1^
Carbohydrate, % energy	39
Fat, % energy	37
Protein, % energy	24

^1^ Hypocaloric balanced diet.

**Table 3:** Subjects’ anthropometric and clinical blood chemistry parameters before and after the HBD in the EHBD group.

Index	Pre-HBD	Post-HBD	*p*	Change	*n*
BW [kg]	82.8 ± 11.0	76.4 ± 9.5	0.000	−6.4 ± 2.5	19
BMI [kg/m^2^]	29.9 ± 2.9	27.6 ± 2.6	0.011	−2.3 ± 0.8	19
WC [cm]	96.9 ± 7.7	90.1 ± 6.8	0.000	−6.8 ± 2.0	19
HC [cm]	103.1 ± 6.2	99.7 ± 6.3	0.070	−3.4 ± 1.9	19
SBP [mmHg]	126.1 ± 11.5	120.0 ± 9.5	0.007	−6.1 ± 8.8	19
DBP [mmHg]	72.3 ± 11.0	68.9 ± 9.2	0.313	−3.3 ± 10.1	19
Albumin [g/L]	45.9 ± 2.1	44.8 ± 2.2	0.027	−1.1 ± 1.9	17
ALT [U/L]	35.8 ± 22.9	22.5 ± 11.6	0.108	−13.3 ± 21.1	19
AST [U/L]	25.2 ± 9.4	26.1 ± 30.7	0.084	0.9 ± 32.1	19
ALP [U/L]	93.1 ± 24.3	83.6 ± 17.6	0.008	−9.4 ± 13.9	19
GGT [U/L]	30.3 ± 17.6	22.2 ± 9.1	0.231	−8.1 ± 15.0	19
BUN [mmol/L]	4.3 ± 1.0	4.5 ± 1.0	0.541	0.2 ± 1.1	19
Creatinine [µmol/L]	74.7 ± 14.4	70.7 ± 13.2	0.002	−3.9 ± 4.8	19
Uric acid [µmol/L]	401.0 ± 110.3	375.0 ± 111.4	0.053	−25.9 ± 54.5	19
TG [mmol/L]	1.5 ± 0.8	1.2 ± 0.5	0.122	−0.3 ± 0.6	19
TC [mmol/L]	4.5 ± 0.9	4.4 ± 0.9	0.515	−0.1 ± 0.7	19
LDL-c [mmol/L]	2.8 ± 0.7	2.6 ± 0.6	0.044	−0.2 ± 0.4	19
HDL-c [mmol/L]	1.0 ± 0.2	1.1 ± 0.3	0.001	0.1 ± 0.1	19
FBG [mmol/L]	5.0 ± 0.6	4.7 ± 0.4	0.029	−0.3 ± 0.5	19
HbA1c [%]	5.1 ± 0.4	5.1 ± 0.4	0.596	0.0 ± 0.3	19

Abbreviations: BW, body weight; BMI, body mass index; WC, waist circumference; HC, hip circumference; SBP, systolic blood pressure; DBP, diastolic blood pressure; ALT, alanine transferase; AST, aspartate transaminase; ALP, alkaline phosphatase; GGT, gamma-glutamyl transpeptidase; BUN, blood urea nitrogen; TG, triglyceride; TC, total cholesterol; LDL-c, low-density lipoprotein cholesterol; HDL-c, high-density lipoprotein cholesterol; FBG, fasting blood glucose; HbA1c, hemoglobin Alc. The *p*-value is calculated using the Wilcoxon rank-sum test or paired *t*-test and represents the difference before and after HBD intervention. The *n-*number is the number of samples before and after the HBD intervention in the EHBD group.

**Table 4:** Subjects’ anthropometric and clinical blood chemistry parameters before and after the HBD in the IHBD group.

Index	Pre-HBD	Post-HBD	*p*	Change	*n*
BW [kg]	93.1 ± 13.4	91.8 ± 13.0	0.001	−1.3 ± 1.7	24
BMI [kg/m^2^]	32.1 ± 3.2	31.6 ± 3.1	0.546	−0.5 ± 0.6	24
WC [cm]	103.9 ± 9.5	102.0 ± 9.4	0.436	−2.4 ± 2.7	24
HC [cm]	106.9 ± 6.3	106.7 ± 6.5	0.949	−0.6 ± 1.6	24
SBP [mmHg]	131.6 ± 11.4	131.3 ± 14.0	0.888	−0.3 ± 11.4	24
DBP [mmHg]	76.6 ± 9.4	75.6 ± 9.1	0.625	−1.0 ± 9.9	24
Albumin [g/L]	46.2 ± 2.6	46.4 ± 2.9	0.675	0.2 ± 2.6	24
ALT [U/L]	54.0 ± 32.0	39.0 ± 19.8	0.132	−15.0 ± 19.4	24
AST [U/L]	31.0 ± 12.4	24.3 ± 7.4	0.030	−6.7 ± 9.0	24
ALP [U/L]	87.2 ± 20.7	87.2 ± 19.0	1.000	0.0 ± 12.1	24
GGT [U/L]	43.5 ± 27.6	42.1 ± 21.6	0.741	−1.4 ± 11.6	24
BUN [mmol/L]	5.6 ± 1.4	6.0 ± 1.5	0.522	0.4 ± 1.3	24
Creatinine [µmol/L]	77.1 ± 12.5	76.9 ± 11.6	0.889	−0.1 ± 5.2	24
Uric acid [µmol/L]	451.5 ± 95.8	442.8 ± 78.5	0.456	−8.7 ± 56.2	24
TG [mmol/L]	2.3 ± 1.4	2.4 ± 1.2	0.749	0.1 ± 1.1	24
TC [mmol/L]	5.1 ± 0.9	5.3 ± 0.8	0.119	0.2 ± 0.6	24
LDL-c [mmol/L]	3.1 ± 1.0	3.2 ± 0.8	0.861	0.0 ± 0.5	24
HDL-c [mmol/L]	0.9 ± 0.2	1.0 ± 0.2	0.014	0.1 ± 0.1	24
FBG [mmol/L]	5.4 ± 0.5	5.2 ± 0.4	0.409	−0.2 ± 0.4	24
HbA1c [%]	5.2 ± 0.3	5.3 ± 0.4	0.156	0.1 ± 0.4	24

Abbreviations: BW, body weight; BMI, body mass index; WC, waist circumference; HC, hip circumference; SBP, systolic blood pressure; DBP, diastolic blood pressure; ALT, alanine transferase; AST, aspartate transaminase; ALP, alkaline phosphatase; GGT, gamma-glutamyl transpeptidase; BUN, blood urea nitrogen; TG, triglyceride; TC, total cholesterol; LDL-c, low-density lipoprotein cholesterol; HDL-c, high-density lipoprotein cholesterol; FBG, fasting blood glucose; HbA1c, hemoglobin Alc. The *p*-value is calculated using the Wilcoxon rank-sum test or paired *t*-test and represents the difference before and after HBD intervention. The *n-*number is the number of samples before and after the HBD intervention in the IHBD group.

**Table 5:** Topological features of the CS0 network and CS12 network in the EHBD group.

Parameter	CF0 Network ^1^	CF12 Network ^2^	*n*
Number of edges	168.000	154.000	19
Number of positive edges	152.000	133.000	19
Number of negative edges	16.000	21.000	19
Number of vertices	114.000	111.000	19
Average degree	2.947	2.775	19
Average clustering coefficient	0.404	0.310	19
Average path length	5.482	3.884	19
Diameter	14.000	12.000	19
Modularity	0.735	0.728	19
Number of modularity	37.000	43.000	19

^1^ before the intervention in the EHBD group; ^2^ after the intervention in the EHBD group. The *n*-number is the number of samples in the EHBD group.

**Table 6:** Topological features of the CF0 network and CF12 network in the IHBD group.

Parameter	CF0 Network ^1^	CF12 Network ^2^	*n*
Number of edges	84.000	86.000	24
Number of positive edges	78.000	77.000	24
Number of negative edges	6.000	9.000	24
Number of vertices	111.000	108.000	24
Average degree	1.514	1.593	24
Average clustering coefficient	0.258	0.484	24
Average path length	3.732	3.472	24
Diameter	10.000	8.000	24
Modularity	0.626	0.815	24
Number of modularity	65.000	63.000	24

^1^ before the intervention in the IHBD group; ^2^ after the intervention in the IHBD group. The *n*-number is the number of samples in the IHBD group.

## Data Availability

Raw sequencing data have been submitted to the National Center for Biotechnology Information (NCBI) under study accession number PRJNA868569. The data presented in this study are available on GitHub: https://github.com/hcwang-jn/gut-HBD (accessed on 26 June 2023).

## Supplementary Materials

The following supporting information can be downloaded at: https://www.mdpi.com/article/10.3390/nu15143101/s1, Figure S1: Differences in the gut microbiota before and after the HBD (in the EHBD and IHBD groups); Figure S2: Differences in the gut microbiota before and after the HBD in the EHBD and IHBD groups; Figure S3: Differences in the gut microbiota before and after the HBD (in the EHBD and IHBD groups).

## References

[1] Abarca-Gómez L., Abdeen Z.A., Hamid Z.A., Abu-Rmeileh N.M., Acosta-Cazares B., Acuin C., Adams R.J., Aekplakorn W., Afsana K., Aguilar-Salinas C.A. (2017). Worldwide trends in body-mass index, underweight, overweight, and obesity from 1975 to 2016: A pooled analysis of 2416 population-based measurement studies in 128·9 million children, adolescents, and adults. Lancet.

[2] Bluher M. (2019). Obesity: Global epidemiology and pathogenesis. Nat. Rev. Endocrinol..

[3] Zeng Q., Li N.S., Pan X.F., Chen L.L., Pan A. (2021). Clinical management and treatment of obesity in China. Lancet Diabetes Endocrinol..

[4] Fan Y., Pedersen O. (2021). Gut microbiota in human metabolic health and disease. Nat. Rev. Microbiol..

[5] Lee C.J., Sears C.L., Maruthur N. (2020). Gut microbiome and its role in obesity and insulin resistance. Ann. N. Y. Acad. Sci..

[6] Gomes A.C., Hoffmann C., Mota J.F. (2018). The human gut microbiota: Metabolism and perspective in obesity. Gut Microbes.

[7] Sonnenburg J.L., Backhed F. (2016). Diet-microbiota interactions as moderators of human metabolism. Nature.

[8] Ji Y., Mao K., Gao J., Chitrakar B., Sadiq F.A., Wang Z., Wu J., Xu C., Sang Y. (2022). Pear pomace soluble dietary fiber ameliorates the negative effects of high-fat diet in mice by regulating the gut microbiota and associated metabolites. Front. Nutr..

[9] Turnbaugh P.J., Ley R.E., Mahowald M.A., Magrini V., Mardis E.R., Gordon J.I. (2006). An obesity-associated gut microbiome with increased capacity for energy harvest. Nature.

[10] Jumpertz R., Le D.S., Turnbaugh P.J., Trinidad C., Bogardus C., Gordon J.I., Krakoff J. (2011). Energy-balance studies reveal associations between gut microbes, caloric load, and nutrient absorption in humans. Am. J. Clin. Nutr..

[11] Yun Y., Kim H.N., Kim S.E., Heo S.G., Chang Y., Ryu S., Shin H., Kim H.L. (2017). Comparative analysis of gut microbiota associated with body mass index in a large Korean cohort. BMC Microbiol..

[12] Sun L.J., Ma L.J., Ma Y.B., Zhang F.M., Zhao C.H., Nie Y.Z. (2018). Insights into the role of gut microbiota in obesity: Pathogenesis, mechanisms, and therapeutic perspectives. Protein Cell.

[13] Gao R., Zhu C., Li H., Yin M., Pan C., Huang L., Kong C., Wang X., Zhang Y., Qu S. (2018). Dysbiosis Signatures of Gut Microbiota Along the Sequence from Healthy, Young Patients to Those with Overweight and Obesity. Obesity.

[14] Schwingshackl L., Zahringer J., Nitschke K., Torbahn G., Lohner S., Kuhn T., Fontana L., Veronese N., Schmucker C., Meerpohl J.J. (2021). Impact of intermittent energy restriction on anthropometric outcomes and intermediate disease markers in patients with overweight and obesity: Systematic review and meta-analyses. Crit. Rev. Food Sci. Nutr..

[15] Headland M.L., Clifton P.M., Keogh J.B. (2019). Correction: Effect of intermittent compared to continuous energy restriction on weight loss and maintenance after 12 months in healthy overweight or obese adults. Int. J. Obes..

[16] Pinto A.M., Bordoli C., Buckner L.P., Kim C., Kaplan P.C., Del Arenal I.M., Jeffcock E.J., Hall W.L. (2020). Intermittent energy restriction is comparable to continuous energy restriction for cardiometabolic health in adults with central obesity: A randomized controlled trial; the Met-IER study. Clin. Nutr..

[17] Parascinet O., Mas S., Hang T., Llavero C., Lorenzo Ó., Ruiz-Tovar J. (2023). A Pilot Study: The Reduction in Fecal Acetate in Obese Patients after Probiotic Administration and Percutaneous Electrical Neurostimulation. Nutrients.

[18] Rodriguez J., Hiel S., Neyrinck A.M., Le Roy T., Potgens S.A., Leyrolle Q., Pachikian B.D., Gianfrancesco M.A., Cani P.D., Paquot N. (2020). Discovery of the gut microbial signature driving the efficacy of prebiotic intervention in obese patients. Gut.

[19] Zhang S., Wu P., Tian Y., Liu B., Huang L., Liu Z., Lin N., Xu N., Ruan Y., Zhang Z. (2021). Gut Microbiota Serves a Predictable Outcome of Short-Term Low-Carbohydrate Diet (LCD) Intervention for Patients with Obesity. Microbiol. Spectr..

[20] Dong T.S., Luu K., Lagishetty V., Sedighian F., Woo S.-L., Dreskin B.W., Katzka W., Chang C., Zhou Y., Arias-Jayo N. (2020). A High Protein Calorie Restriction Diet Alters the Gut Microbiome in Obesity. Nutrients.

[21] Meslier V., Laiola M., Roager H.M., De Filippis F., Roume H., Quinquis B., Giacco R., Mennella I., Ferracane R., Pons N. (2020). Mediterranean diet intervention in overweight and obese subjects lowers plasma cholesterol and causes changes in the gut microbiome and metabolome independently of energy intake. Gut.

[22] Deledda A., Palmas V., Heidrich V., Fosci M., Lombardo M., Cambarau G., Lai A., Melis M., Loi E., Loviselli A. (2022). Dynamics of Gut Microbiota and Clinical Variables after Ketogenic and Mediterranean Diets in Drug-Naïve Patients with Type 2 Diabetes Mellitus and Obesity. Metabolites.

[23] Basciani S., Camajani E., Contini S., Persichetti A., Risi R., Bertoldi L., Strigari L., Prossomariti G., Watanabe M., Mariani S. (2020). Very-Low-Calorie Ketogenic Diets with Whey, Vegetable, or Animal Protein in Patients with Obesity: A Randomized Pilot Study. J. Clin. Endocrinol. Metab..

[24] Zeevi D., Korem T., Zmora N., Israeli D., Rothschild D., Weinberger A., Ben-Yacov O., Lador D., Avnit-Sagi T., Lotan-Pompan M. (2015). Personalized Nutrition by Prediction of Glycemic Responses. Cell.

[25] Yuan W., Lu W., Wang H., Wu W., Zhou Q., Chen Y., Lee Y.K., Zhao J., Zhang H., Chen W. (2021). A multiphase dietetic protocol incorporating an improved ketogenic diet enhances weight loss and alters the gut microbiome of obese people. Int. J. Food Sci. Nutr..

[26] Wickham H. (2009). ggplot2: Elegant Graphics for Data Analysis.

[27] Ahlmann-Eltze C., Patil I. (2021). ggsignif: R Package for Displaying Significance Brackets for ‘ggplot2’. https://psyarxiv.com/7awm6/.

[28] Dray S., Dufour A.-B. (2007). The ade4 Package: Implementing the Duality Diagram for Ecologists. J. Stat. Softw..

[29] Revelle W. (2013). psych: Procedures for Psychological, Psychometric, and Personality Research. https://www.researchgate.net/publication/281345624_psych_Procedures_for_Psychological_Psychometric_and_Personality_Research_R_Package_Version_10-95.

[30] Dixon P. (2003). VEGAN, a Package of R Functions for Community Ecology. J. Veg. Sci..

[31] Segata N., Izard J., Waldron L., Gevers D., Miropolsky L., Garrett W.S., Huttenhower C. (2011). Metagenomic biomarker discovery and explanation. Genome Biol..

[32] Benjamini Y., Krieger A.M., Yekutieli D. (2006). Adaptive linear step-up procedures that control the false discovery rate. Biometrika.

[33] Langfelder P., Horvath S. (2012). Fast R Functions for Robust Correlations and Hierarchical Clustering. J. Stat. Softw..

[34] Bastian M., Heymann S., Jacomy M. Gephi: An Open Source Software for Exploring and Manipulating Networks. Proceedings of the Third International Conference on Weblogs and Social Media, ICWSM 2009.

[35] Pedregosa F., Varoquaux G., Gramfort A., Michel V., Thirion B., Grisel O., Blondel M., Prettenhofer P., Weiss R., Dubourg V. (2011). Scikit-learn: Machine Learning in Python. J. Mach. Learn. Res..

[36] Chen T., Guestrin C. XGBoost: A Scalable Tree Boosting System. Proceedings of the KDD ‘16: Proceedings of the 22nd ACM SIGKDD International Conference on Knowledge Discovery and Data Mining.

[37] Ke G., Meng Q., Finley T., Wang T., Chen W., Ma W., Ye Q., Liu T.-Y. Lightgbm: A highly efficient gradient boosting decision tree. Proceedings of the 31st International Conference on Neural Information Processing Systems.

[38] Ozato N., Saito S., Yamaguchi T., Katashima M., Tokuda I., Sawada K., Katsuragi Y., Kakuta M., Imoto S., Ihara K. (2019). Blautia genus associated with visceral fat accumulation in adults 20–76 years of age. NPJ Biofilms Microbiomes.

[39] Okyere S.K., Wen J., Cui Y., Xie L., Gao P., Zhang M., Wang J., Wang S., Ran Y., Ren Z. (2022). *Bacillus toyonensis* SAU-19 and SAU-20 Isolated from *Ageratina adenophora* Alleviates the Intestinal Structure and Integrity Damage Associated with Gut Dysbiosis in Mice Fed High Fat Diet. Front. Microbiol..

[40] Sbierski-Kind J., Grenkowitz S., Schlickeiser S., Sandforth A., Friedrich M., Kunkel D., Glauben R., Brachs S., Mai K., Thürmer A. (2022). Effects of caloric restriction on the gut microbiome are linked with immune senescence. Microbiome.

[41] Stanislawski M.A., Frank D.N., Borengasser S.J., Ostendorf D.M., Ir D., Jambal P., Bing K., Wayland L., Siebert J.C., Bessesen D.H. (2021). The Gut Microbiota during a Behavioral Weight Loss Intervention. Nutrients.

[42] Ott B., Skurk T., Hastreiter L., Lagkouvardos I., Fischer S., Buttner J., Kellerer T., Clavel T., Rychlik M., Haller D. (2017). Effect of caloric restriction on gut permeability, inflammation markers, and fecal microbiota in obese women. Sci. Rep..

[43] Ottosson F., Brunkwall L., Ericson U., Nilsson P.M., Almgren P., Fernandez C., Melander O., Orho-Melander M. (2018). Connection Between BMI-Related Plasma Metabolite Profile and Gut Microbiota. J. Clin. Endocrinol. Metab..

[44] Hosomi K., Saito M., Park J., Murakami H., Shibata N., Ando M., Nagatake T., Konishi K., Ohno H., Tanisawa K. (2022). Oral administration of *Blautia wexlerae* ameliorates obesity and type 2 diabetes via metabolic remodeling of the gut microbiota. Nat. Commun..

[45] Odamaki T., Kato K., Sugahara H., Hashikura N., Takahashi S., Xiao J.Z., Abe F., Osawa R. (2016). Age-related changes in gut microbiota composition from newborn to centenarian: A cross-sectional study. BMC Microbiol..

[46] Dong T.S., Luu K., Lagishetty V., Sedighian F., Woo S.L., Dreskin B.W., Katzka W., Chang C., Zhou Y., Arias-Jayo N. (2021). The Intestinal Microbiome Predicts Weight Loss on a Calorie-Restricted Diet and Is Associated With Improved Hepatic Steatosis. Front. Nutr..

[47] Crost E.H., Coletto E., Bell A., Juge N. (2023). *Ruminococcus gnavus*: Friend or foe for human health. FEMS Microbiol. Rev..

[48] Bell A., Brunt J., Crost E., Vaux L., Nepravishta R., Owen C.D., Latousakis D., Xiao A., Li W., Chen X. (2019). Elucidation of a sialic acid metabolism pathway in mucus-foraging *Ruminococcus gnavus* unravels mechanisms of bacterial adaptation to the gut. Nat. Microbiol..

[49] Jie Z., Yu X., Liu Y., Sun L., Chen P., Ding Q., Gao Y., Zhang X., Yu M., Liu Y. (2021). The Baseline Gut Microbiota Directs Dieting-Induced Weight Loss Trajectories. Gastroenterology.

[50] Lozano C.P., Wilkens L.R., Shvetsov Y.B., Maskarinec G., Park S.Y., Shepherd J.A., Boushey C.J., Hebert J.R., Wirth M.D., Ernst T. (2022). Associations of the Dietary Inflammatory Index with total adiposity and ectopic fat through the gut microbiota, LPS, and C-reactive protein in the Multiethnic Cohort-Adiposity Phenotype Study. Am. J. Clin. Nutr..

[51] Yan H., Qin Q., Chen J., Yan S., Li T., Gao X., Yang Y., Li A., Ding S. (2021). Gut Microbiome Alterations in Patients with Visceral Obesity Based on Quantitative Computed Tomography. Front. Cell. Infect. Microbiol..

[52] Lin Y., Bai M., Wang S., Chen L., Li Z., Li C., Cao P., Chen Y. (2022). Lactate Is a Key Mediator That Links Obesity to Insulin Resistance via Modulating Cytokine Production from Adipose Tissue. Diabetes.

[53] Chen L.M., Collij V., Jaeger M., van den Munckhof I.C.L., Vila A.V., Kurilshikov A., Gacesa R., Sinha T., Oosting M., Joosten L.A.B. (2020). Gut microbial co-abundance networks show specificity in inflammatory bowel disease and obesity. Nat. Commun..

[54] Hall A.B., Yassour M., Sauk J., Garner A., Jiang X.F., Arthur T., Lagoudas G.K., Vatanen T., Fornelos N., Wilson R. (2017). A novel *Ruminococcus gnavus* clade enriched in inflammatory bowel disease patients. Genome Med..

