# Supplementary figures and images for: Modulating the Human Gut Microbiota through Hypocaloric Balanced Diets: An Effective Approach for Managing Obesity
Source: Nutrients. 2023 Jul 11;15(14):3101. doi: 10.3390/nu15143101 (PMC10383620; doi:10.3390/nu15143101)

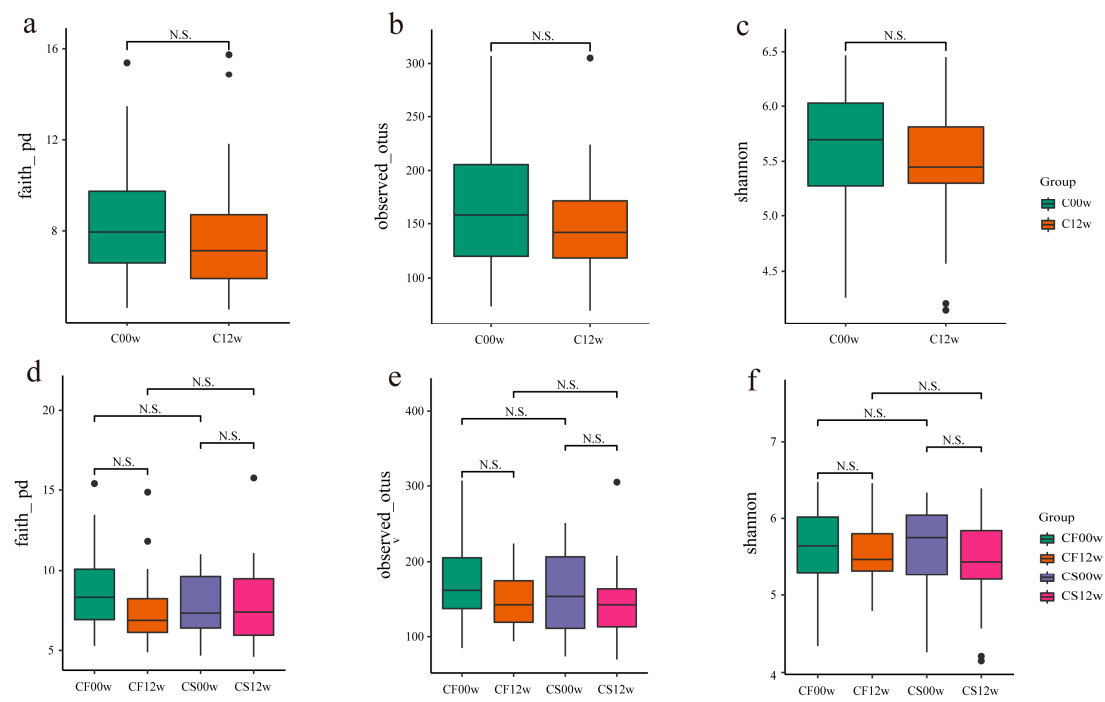

**Figure S1.** Differences in the gut microbiota before and after the HBD (in the EHBD and IHBD groups).

Supplement: Supplementary file 1 [file nutrients-15-03101-s001.zip › Figure S1.pdf]

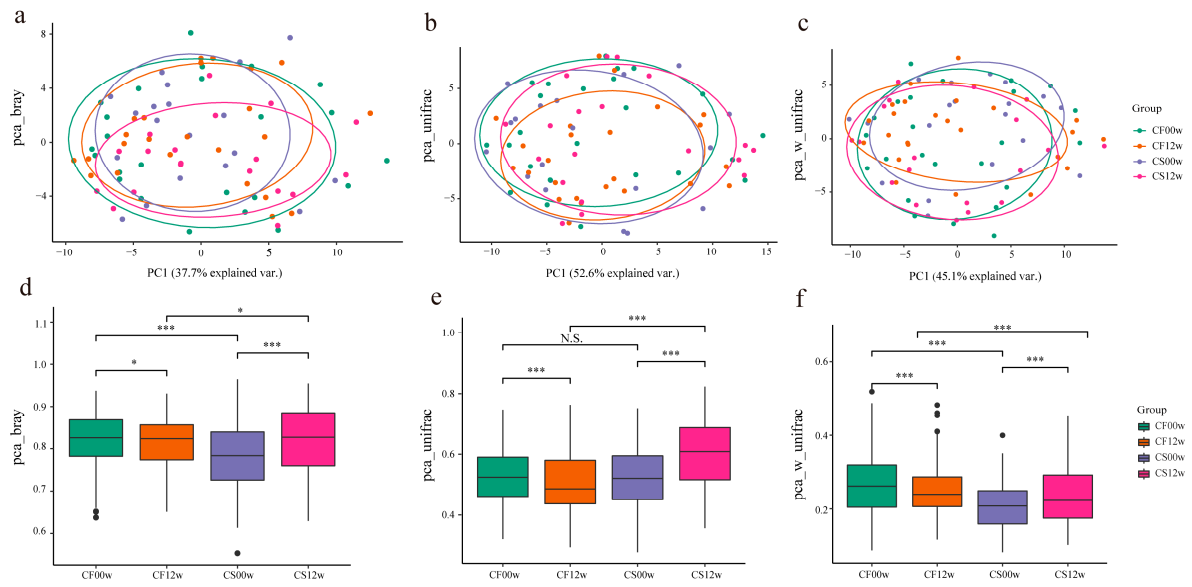

**Figure S2.** Differences in the gut microbiota before and after the HBD in the EHBD and IHBD groups.

Supplement: Supplementary file 1 [file nutrients-15-03101-s001.zip › Figure S2.pdf]

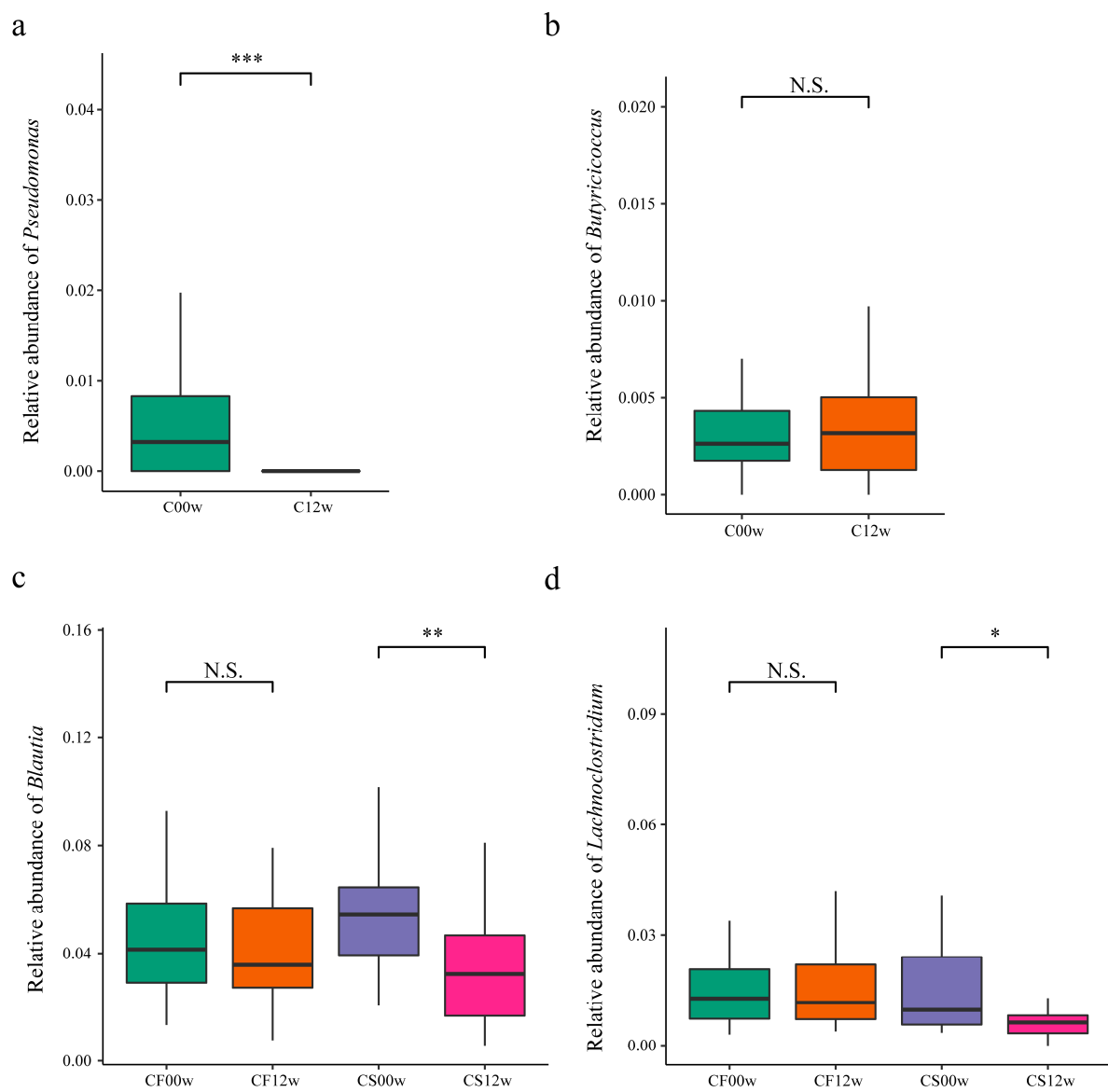

**Figure S3.** Differences in the gut microbiota before and after the HBD (in the EHBD and IHBD groups).

Supplement: Supplementary file 1 [file nutrients-15-03101-s001.zip › Figure S3.pdf]
